# Wave-Induced Flow in Meridians Demonstrated Using Photoluminescent Bioceramic Material on Acupuncture Points

**DOI:** 10.1155/2013/739293

**Published:** 2013-11-07

**Authors:** C. Will Chen, Chen-Jei Tai, Cheuk-Sing Choy, Chau-Yun Hsu, Shoei-Loong Lin, Wing P. Chan, Han-Sun Chiang, Chang-An Chen, Ting-Kai Leung

**Affiliations:** ^1^Department of Bioengineering, Tatung University, No. 40, Sec. 3, Zhongshan N. Road, Taipei 104, Taiwan; ^2^Department of Traditional Chinese Medicine, Taipei Medical University Hospital, No. 252, Wu Hsing Street, Taipei 110, Taiwan; ^3^Department of Medicine, Taipei Medical University, No. 250, Wu Hsing Street, Taipei 110-52, Taiwan; ^4^Emergency and Intensive Care Department, Taipei Hospital, Department of Health, No. 127, Su Yuan Road, Hsinchuang, New Taipei City 242-13, Taiwan; ^5^Graduated Institute of Communication Engineering, Tatung University, No. 40, Sec. 3, Zhongshan N. Road, Taipei 104, Taiwan; ^6^Department of Surgery, Taipei Hospital, Ministry of Health and Welfare, No. 127, Su Yuan Road, Hsinchuang, New Taipei City 242-13, Taiwan; ^7^Department of Surgery, School of Medicine, College of Medicine, Taipei Medical University, No. 250, Wu Hsing Street, Taipei 110-52, Taiwan; ^8^Department of Diagnostic Radiology, Taipei Municipal Wanfang Hospital, No. 111, Sec. 3, Hsing Long Road, Taipei 116, Taiwan; ^9^Department of Radiology, School of Medicine, College of Medicine, Taipei Medical University, No. 250, Wu Hsing Street, Taipei 110-52, Taiwan; ^10^College of Medicine, Fu Jen Catholic University, No. 510, Zhongzheng Road, Xinzhuang, New Taipei City 24205, Taiwan; ^11^Graduate Institute of Mechanical and Electrical Engineering, National Taipei University of Technology, No. 1, Sec. 3, Zhongxiao E. Road, Taipei 10608, Taiwan; ^12^Department of Physics & College of Science and Engineering, Fu Jen Catholic University, No. 510, Zhongzheng Road, Xinzhuang, New Taipei City 24205, Taiwan; ^13^Department of Radiology, Taipei Hospital, Ministry of Health and Welfare, No. 127 Su Yuan Road, Hsinchuang, New Taipei City 242-13, Taiwan

## Abstract

The mechanisms of acupuncture remain poorly understood, but it is generally assumed that measuring the electrical conductivity at various meridians provides data representing various meridian energies. In the past, noninvasive methods have been used to stimulate the acupuncture points at meridians, such as heat, electricity, magnets, and lasers. Photoluminescent bioceramic (PLB) material has been proven to weaken hydrogen bonds and alter the characteristics of liquid water. In this study, we applied the noninvasive PLB technique to acupuncture point irradiation, attempting to detect its effects by using electrical conductivity measurements. We reviewed relevant literature, searching for information on meridians including their wave-induced flow characteristics.

## 1. Introduction

Acupuncture meridians are traditionally believed to comprise channels that connect the surface of the body with internal organs. Twelve primary meridians are believed to be located bilaterally in the body. The left and right meridians are symmetrical and influence each other through the interconnected meridian channels [1–4]. A network of meridian channels is believed to be located within soft connective tissues. Traditional Chinese medicine (TCM) has extensively described the related normal physiological functions, pathological conditions, transmission of the senses, and possible disease mechanisms [5]. The mechanisms of acupuncture remain poorly understood and require further investigation with scientific methods to explore the nature of meridian lines and acupuncture points. It is generally assumed that measuring the electrical conductivity at various meridians provides data relevant to the so called “meridian energy” [1].

In 1950, Dr. Yoshio Nakatani measured the electrical resistance of the skin of his patients, revealing poor electrical conductivity. He discovered that some patients presented excessive skin resistance. Because this line exhibited the increased electrical conductivity and seemed to follow a traditional meridian, he named it the “Ryodoraku,” meaning a strong conductive line or electrical pathway. The Ryodoraku lines were defined as they corresponded to various classical Chinese meridian pathways [6].

In this study, we used Ryodoraku equipment to measure meridian current flow by using electrodermal measurement on the Ryodoraku meridian points. Many previous studies have measured current levels by applying Ryodoraku techniques [1, 6–10]. The 24 measured Ryodoraku meridian points are located on the twelve primary meridians; thus, based on the theories of TCM, the resulting electrodermal measurements are regarded as measurements of meridian energy [1].

A previous study of meridian energy or current flow suggested that specific diseases may be related to more than a single meridian [1] (e.g., gastric diseases to the stomach). A nonspecific relationship exists between abnormal organic findings and the corresponding meridian energies. These results support the hypothesis that all meridians are interconnected [1–4].

During the traditional acupuncture, a needle penetrates the dermis of the skin and subcutaneous tissue. It then passes through interstitial connective tissues, potentially reaching deeper structures such as muscles, nerves, bones, or other vital organs. Although adverse reactions to traditional acupuncture are rare, complications such as regional subcutaneous or systemic infections, neuropathy, spinal injuries, pneumothorax, and cardiac injury are occasionally reported [11, 12]. In this study, we used a non-invasive technique of PLB to irradiate acupuncture points and applied electrodermal measurement to assess whether PLB reinforces the effects of needle acupuncture on meridians. It was previously proven that PLB weakens hydrogen bonds and alters the characteristics of liquid water [13, 14]. In this study, we applied the PLB technique to the Ryodoraku meridian point irradiation, attempting to detect its effects on the meridian current flow by the possible alteration of liquid characteristics in the meridian channels; thus, this study may demonstrate the fluid-wave characteristics of meridians [13, 14]. In the past, noninvasive methods have been used to stimulate the acupuncture points, such as heat, electricity, magnets, and lasers, but none of these can replace the traditional acupuncture needles. Using PLB acupuncture point irradiation is painless, nontraumatic, and poses no risk of infection; this study should demonstrate the advantages of the PLB method [15]. We reviewed relevant research, to support our hypothesis that PLB irradiation is suitable for application on meridians.

## 2. Materials and Methods

### 2.1. Participants

The participants were adults who consented to participate in this trial offered by the Departments of Traditional Medicine and Diagnostic Radiology, from March 2013 to June 2013. There are totally 147 meridian points on different candidates that were involved in this study. The study was approved by the human subjects committee at the hospital (approval number: TMU-JIRB201207024).

### 2.2. Measurements

**Figure 1:**
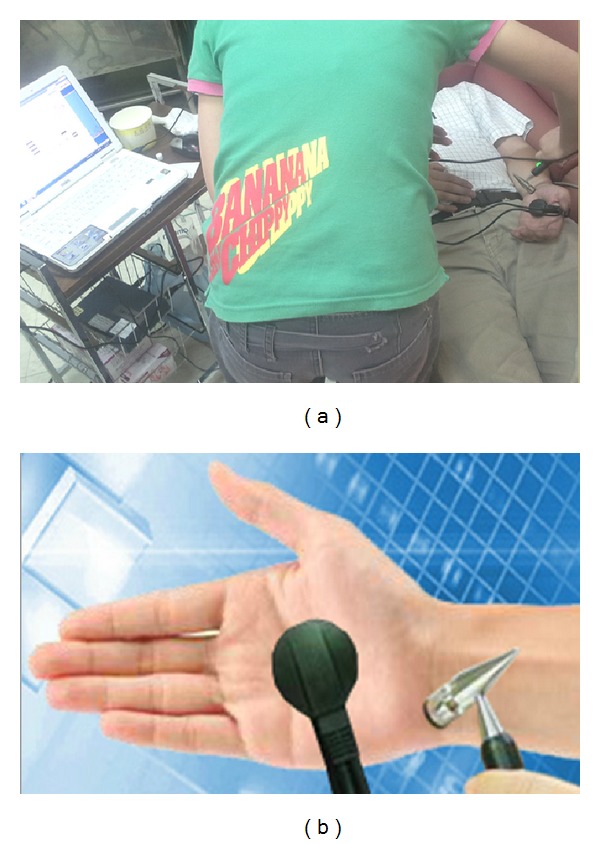
Electrodermal measurements of various acupuncture points on the meridian line (a), using a metal cylinder, electrode, and spring-loaded probe (b).

We measured the meridian current by using a MEAD Me-Pro, 6th generation (Hanja International CO. Ltd., Taoyuan, Taiwan) device, which yielded electrodermal measurements of the 24 Ryodoraku meridian points (lung (LU9), pericardium (PC7), heart (HT7), small intestine (SI4),triple energizer (SJ4), large intestine (LI5), spleen (SP3), liver (LR3), kidney (KI4), bladder (BL65), gallbladder (GB40), and stomach (ST42)) and was similar to the equipment used in previous studies [1, 7–9]. The MEAD device was designed based on the Ryodoraku theory. The machine comprises two electrodes: the first, a metal cylinder held in the left hand of the patient, and the second, connected to a spring-loaded probe containing cotton moistened with physiological saline solution. A trained technician applies (Figure 1) the second electrode to the 24 acupuncture points along the 12 meridians (12 left plus 12 right). The measurements begin using a low current, which is probably gradually increased to a maximal value of 200 *μ*A. The electrical conductivity readings for the meridian points are recorded into a computerized system. The participants with normal or abnormal meridian current findings are divided into six categories: extremely high current level, moderately high current level, normally high current level, extremely low current level, moderately low current level, and normally low current level.

This MEAD device is advantageous because the overall current levels remain in good repeatability when the device is operated by a trained technician (with at least 60 minutes of training or past examinations of at least 10 patients). According to the results of the mean overall meridian current, the technician selected the most abnormal current level on meridian channels and its specific Ryodoraku meridian points. The lowest current level meridian measurement (LCLMM) and highest current level meridian measurement (HCLMM) were both selected.

### 2.3. Photoluminescent Bioceramic Materials

**Figure 2:**
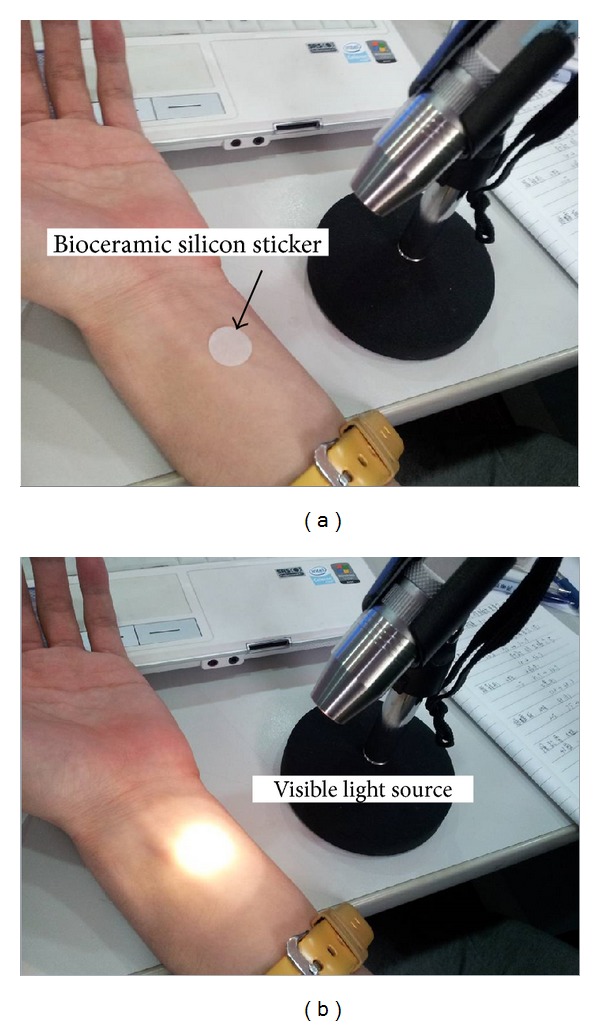
PLB irradiation on acupuncture points and remeasurement.

Ceramic powder was obtained from the laboratory of radiology of Taipei Medical University (Taipei, Taiwan). The bioceramic material consisted of microsized particles produced from various elemental components [13, 14, 16–29]. Of the bioceramic material, 7% was embedded in a silicon sticker with good translucence (YY Rubber Company, Foshan, Guangdong, PRC). Photoluminescence is a special type of luminescence, referring to materials that absorb light energy and then release that energy in the form of light; it also describes the interaction between electromagnetic radiation and matter. The PLB of this bioceramic material absorbs some specific wavelength spectrum (including near, middle, and far infrared) and was provided using visible light source irradiation, which was directed to the silicon sticker placed on the selected meridian lines at the corresponding acupuncture point (Figure 2). The light sources were visible light-emitting diodes (LEDs), which emitted wavelengths of a visible white light spectrum between 480 nm and 780 nm. We strictly controlled the level of illumination at 450 lux ± 50 lux, avoiding thermal effects on the skin of the participants.

### 2.4. To Analyze Possible Effects on Meridian Points on Different Candidates

**Figure 3:**
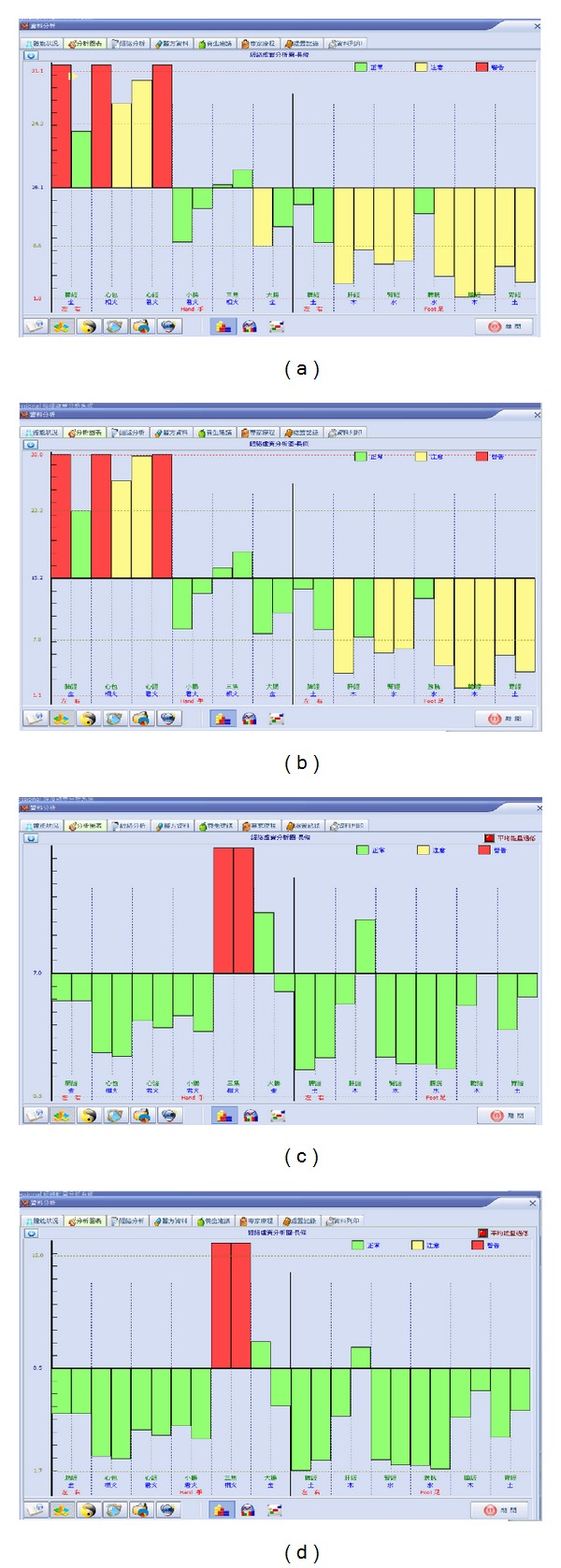
(a, c) Two examples of candidates on instrumental measurements; (b, d) the repeated measurements (within 15 minutes) showed good repeatability.

The participants with meridian current findings (Figure 3) were divided into six categories: extremely high current level, moderately high current level, normally high current level, extremely low current level, moderately low current level, and normally low current level. After selecting the most abnormal current levels on meridian lines (LCLMM or HCLMM) and their corresponding acupuncture points for each patient, we performed PLB irradiation for 15 minutes (Figure 2). The meridian current was reexamined by using the MEAD Me-Pro, which yielded new electrodermal measurements of the 24 meridians. The second result of the overall mean of meridian currents was compared with the previous measurements from 15 minutes prior.

We calculated and compared before and after PLB irradiations on the mean current levels of each candidate the normalizing ability of PLB irradiations on extremely high and moderately high current level group and also on extremely low and moderately low current level group on different meridian channels.

### 2.5. To Observe the Possibility of One Specific Meridian Channel Indirectly to Affect Another Meridian Channel (Crossover Effect)

After PLB treatment of one specific meridian channel (e.g., gall bladder, lung or triple energizer), we observe that the current level of another specific meridian channel could be influenced. We supposed the specific meridian channel is being influenced as (1) change of current level over 50%; (2) change of its current level category (six categories of extremely high current level, moderately high current level, normally high current level, extremely low current level, moderately low current level, and normally low current level). In other word, the influence is defined as positive “crossover” effect by PLB treatment.

### 2.6. Data Analysis

The 2006 version of AutoCAD was used for data processing, calculating the percentage change in the scale and ratio of the current levels at various meridian channels before and after the PLB irradiation of acupuncture points. Statistical analyses were performed using SPSS 15.0 software (SPSS Inc., Chicago, Ill, USA). We examined the individual variables by using percentages, means, and standard deviations, exploring group differences by using paired samples *t*-test.

### 2.7. Literature Research

Using Google Scholar (http://scholar.google.com.tw/), we searched for relevant literature published from 2000 to 2013, using keywords combinations such as *meridian, acupuncture, propagation, stimulation, current, water, fluid, anatomy, energy, transport, flow*, and *propagation*. We determined the total number of citations in each subject in the study period and selected the major research articles (in English) to further our discussion. Moreover, critical findings are presented when electromagnetic methods and radiological images are used to evaluate the anatomy and characteristics of meridians.

## 3. Results

### 3.1. Repeatable Results before PLB Irradiation

As previously mentioned, the MEAD Me-Pro maintains stable levels of current; a trained technician can easily repeat the results of a test (Figure 3).

### 3.2. Overall Meridian Current

**Figure 4:**
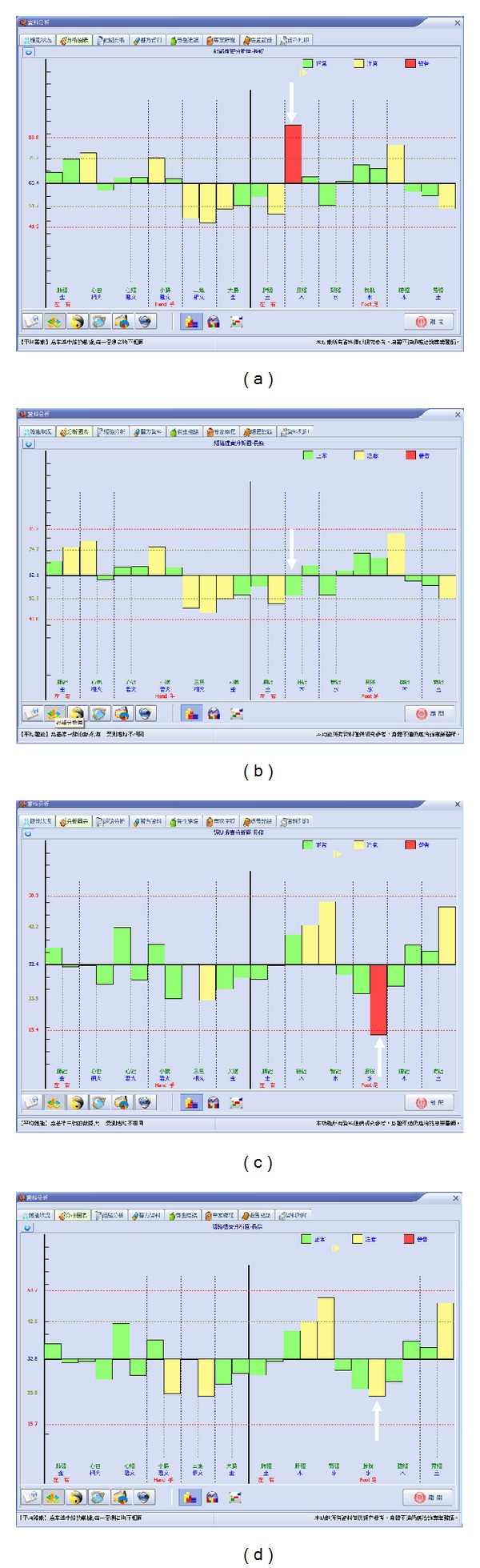
(a, c) Two examples of candidates with extremely high and low current levels measured by instrumental measurements; (b, d) after PLB irradiation of 15 minutes, there are remarkable normalization of current levels on the two specific meridian channels.

**Figure 5:**
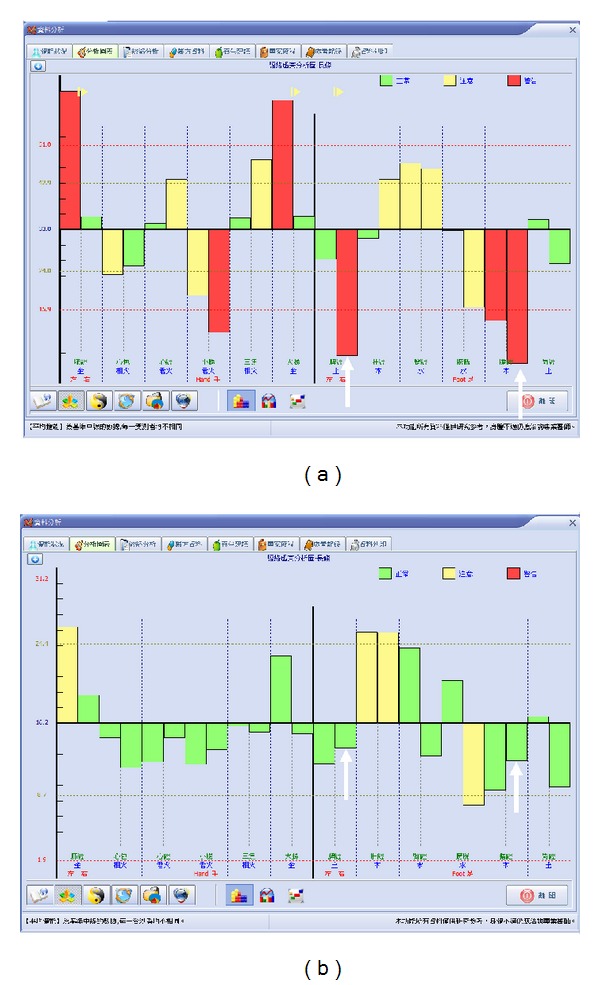
(a) One example of candidate with six different extremely high and low current levels of meridian channels; (b) After PLB irradiation of corresponding acupuncture points of the two of the abnormal meridian channels, there are remarkable normalization of current levels on all of the six specific meridian channels.

The participants with abnormal meridian current findings such as extremely high current level or extremely low current level received PLB irradiation on the specific corresponding acupuncture points and then remeasured after 15 minutes. The results showed significant effect and tendency of their normalization of abnormal meridian currents (Figure 4). Besides, there is also observed tendency of interaction between specific meridian channels (Figure 5). There are totally 147 meridian points on 76 candidates that received PLB irradiations on specific corresponding acupuncture points. Amongst them, 69 meridian points were measured as extremely high or moderately high current levels. There were 78 meridian points measured as extremely low or moderately low current levels.

### 3.3. Results of Mean Current Level Change and Normalizing Ability after PLB Irradiations

Table 1 shows that there is no significant difference (*P*-value = 0.054) of the candidates (*n* = 76) after PLB irradiations on their mean current levels (averaging from their 24 Ryodoraku meridian points). But there are significant normalizing ability of PLB irradiations on extremely high and moderately high current level group (*n* = 69; positive current values above the mean current level); and on extremely low and moderately low current level group (*n* = 78; negative current values below the mean current level). Therefore, the PLB irradiation significantly regulated the meridian points and tended to normalize the current flow of abnormal meridians approaching their normal current level.

### 3.4. Results of One Specific Meridian Channel Indirectly to Affect Another Meridian Channel (Crossover Effect) 

There is about 73.7% (56/76) of testing candidates that revealed “crossover” influence of one specific meridian to another meridian channels such as “lung,” “liver,” “gall bladder,” “spleen,” “triple energizer,” “heart,” and “pericardium.” Among the positive “crossover” effect (change of current level over 50% or change of its current level category) of meridian by PLB treatment is the following, (the most explicit findings on PLB treatment in decreasing order) (Table 2): “lung” to affect “liver” (86%) > “gall bladder” to affect “lung” (71%) = “lung” to affect “spleen” (71%) > “triple energizer” to affect “spleen” (60%) > “gall Bladder” to affect “pericardium” (54%) > “gall Bladder” to affect “heart” (50%). Therefore, the PLB irradiation had a tendency to provoke intermeridian interaction.

### 3.5. Review of Literature

We located scant publications related to the topic of the current study. We selected a time range (2000–2013) and the English language, searching for studies regarding electromagnetic methods, radiological images, and the characteristics of meridians.  Table 3 lists the papers selected for discussion.

## 4. Discussion

In Table 1, we prove that the 15 minutes of PLB irradiation exhibits a reliable complementary effect on LCLMM, rehabilitating to or approaching the normal current level; a 15-minute PLB irradiation also tended to suppress the HCLMM.

**Figure 6:**
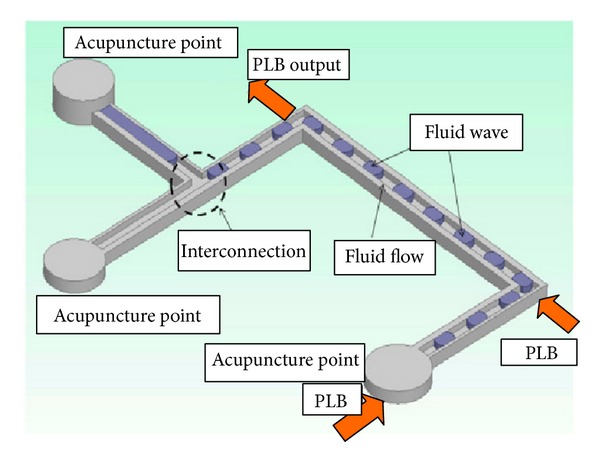
PLB irradiation of the specific meridian (acupuncture) points may induce a wave-induced flow in the meridians, by using the interconnection of various meridians.

In Table 2, we show that the specific meridian channel current is indirectly affected by another meridian channel which has been treated by the PLB irradiation. Because the meridian channels and their corresponding acupuncture points are located in distinct locations, typical light energy irradiation should not be able to affect the electrical resistance of the skin or other meridian channels if no interconnecting network exists. Our previous water-based experiment proved that PLB weakens the hydrogen bonds and modifies the characteristics of liquid water [13, 14]. In this study, we applied the PLB technique to the Ryodoraku meridian point irradiation, attempting to detect its effects on the meridian current flow by the possible alteration of liquid characteristics in the meridian channels. Table 1 (Figure 4 as one example) shows that PLB irradiation has complementary effects on the current flow of abnormal meridians returning to its normal current level. Table 2 (Figure 5 as one example) shows that there are evident interactions between the current flows of relative meridians. Based on results of the current study, we suggest that the meridian channels are interconnected and acupuncture point stimulation induces a systematic wave-induced flow as shown in Figure 6.

To strengthen our hypothesis, we searched for support in the relevant literature. Although numerous studies of TCM are published each year, the results and corresponding discussions are typically TCM theories, which are rarely proven through scientific methods. In order to decrease the amount of bias and avoid referring to unproven TCM theories, our discussion is based exclusively on the results of evidence-based publications. 

After reviewing recent studies of meridians, acupuncture, propagation, stimulation, current, water, fluid, anatomy, energy, transport, flow, and propagation (Table 3), we discuss the progression of scientific evidence regarding meridians and the possibility of wave-induced flows.

### 4.1. Current and Low Impedance Characteristics of Meridians

After using a single-power alternating current (SPAC) instrument to measure low-impedance acupuncture points, it was determined that the mean subcutaneous impedance at the acupuncture points was significantly lower than it was at the impedance of control points; subcutaneous impedance was lower at the low-impedance points measured using the SPAC two-electrode method. This suggests that a high amount of interstitial fluid lies beneath the low-impedance acupuncture points [30]. Previous studies have suggested that the acupuncture meridians are physiologically characterized by low electrical impedance and anatomically associated with the planes of connective tissue. 

### 4.2. Anatomy of Meridians

Regarding the possible location of meridians, previous publications have suggested collagenous bands and the fascial plane. Collagenous bands, which can be detected by increasing the echogenicity of an ultrasound, are significantly associated with lower electrical impedance and may explain the reduced impedance that was previously reported at the acupuncture meridians. This finding provides critical insights about acupuncture meridians and the relevance of collagen in bioelectrical measurements. Acupuncture points are likely located on the skin overlying the fascial planes that separate muscles; thus, acupuncture meridians may be located along the fascial planes between muscles or between a muscle and bone or tendon [5, 32]. Magnetic resonance imaging suggests that acupuncture points are located at connective tissue sites and cleavage planes [31].

### 4.3. Energy Consumption of Meridians

When a highly sensitive CO_2_ instrument was used to measure the transcutaneous CO_2_ emissions at the meridian lines, it showed that the level of the emission was highly related to the positions of acupuncture points and meridian lines on the body. It was concluded that a strong correlation exists in energy metabolism activity among the body surfaces along the meridian [38]. After moxibustion (or similar light stimulation) of the body in the 3 *μ*m–5 *μ*m range, light channels appear on the body, demonstrating the existence of the acupuncture meridian structure.

It was proven that high temperature responses can occur along the meridians in physiological and pathological conditions, suggesting that meridians have infrared or near infrared radiation characteristics. These findings appear to confirm the existence of acupuncture meridians, suggesting that living matter is not in the ground state, but rather permanently excited [35, 41].

### 4.4. Light Propagation of Meridians

Previous studies have used non-invasive methods to detect the human meridian system. When the optical transport properties of visible laser lights and halogen lamps were used to irradiate meridian and nonmeridian pathways, it was suggested that the optical properties of the human meridian significantly differ from the surrounding tissues [42, 43]. The study concluded that the strong light propagation and optical properties along the meridian channel comprised a histological structure correlated with interstitial fluids [42, 43]. 

### 4.5. Radioactive Isotopes Pass through the Meridian Channel

Numerous experiments have proven that a radioactive tracer inserted at an acupuncture point follows a course corresponding to the meridians described by TCM. According to human anatomy, these pathways are neither part of the vascular system, nor the lymphatic ducts, and the velocity of the radioactive message suggests that they are not transferred along the nervous system. Thus, the meridian channels are likely individual pathways, separated from the microcirculation, vessels, lymphatic ducts, and nervous system [36, 37, 39, 44–47].

### 4.6. Flow Channel Characteristics of Meridians

A hydrodynamic analysis of the waveforms stimulated by vibration stimuli at meridian and nonmeridian points was conducted by using the optimal stimulator frequency at the pericardium meridian. It was determined that the mean transfer speed in the meridian was significantly lower than in the adjacent control region, and differences in the attenuation rate and peak amplitude were also noted [39]. Zhang et al. [40] conducted a hydromechanic study, exploring the fundamentals of acupuncture points and meridians, and measuring the transmission of artificial interstitial fluid pressure waves to examine their connection with the low resistance points; a strong connection was confirmed between the points. This indicates that the points form channels along the meridians (low-hydraulic resistance channels), corresponding with the meridian channels described in TCM. Interstitial fluid is an essential body fluid, which connects blood vessels, lymphatic ducts, and intracellular spaces; however, modern physiology pays little attention to interstitial fluid, and some clinicians debate whether interstitial fluid actually flows freely [48–50]. Their results showed that a lower hydraulic resistance channels (LHRC) existed along the meridians. The discovery of LHRCs provides the first physiological explanation for meridians, and the flow channel could interpret as the movement of isotope tracks. Another human study using an isotope tracing method showed that isotopes migrate along the meridian lines, deducing that this movement represented the flow of interstitial fluid along the LHRCs. Combining Zhang et al. [40] and other findings confirms that the meridian channels exist among the subcutaneous tissues and demonstrate the characteristics of fluid flow [51–61].

### 4.7. Contributions and Limitations

In our opinion, meridian lines are interstitial microscopic fluid channels and fulfilling most of the previously mentioned characteristics [62]. Although pure water containing no electrolytes or ions is an excellent electrical insulator, water is an effective solvent and always contains some dissolved solutes such as sodium chloride or other salts; water containing few impurities is a strong conductor of electricity [63–65]. In typical circumstances, water is able to propagate or transfer sound [66], visible light [67], heat (infrared) [68], and radioactive isotopes [69].

Based on the results of the current study and our review of the literature, we suggest that the hydrodynamic of waveforms fluid flow and interstitial fluid concepts [70] of the meridians and acupuncture points explains the reported transmission of current [30, 32], acoustic responses [33, 71], thermal responses [34, 35], optical transmissions [34, 43], isotope passages [36, 37, 72], hydrodynamic analysis [40, 73], and PLB stimulation [13, 14] in meridians. The hypothesis that meridians are open channels of interstitial fluid seems to be accepted, based on evidence-based research. Some limitations must be considered. In the future, we plan to create methods of observing and measuring the wave movement pattern and direction of induced flow within the meridian channels (Figure 6). Demonstrating the objective existence and 3D network of meridians requires combining various technologies including biophysics, biochemistry, molecular biology, and radiological imaging. 

## 5. Conclusion

In this study, we applied the PLB technique to the Ryodoraku meridian point irradiation, attempting to detect its effects on the meridian current flow by the possible alteration of liquid characteristics in the meridian channels. Our data show that PLB has complementary effects for current flow of abnormal meridians returning to its normal current level, and there are significant interactions between the current flows of relative meridians. In the future PLB can be used to regulate meridian current flow and provoke the intermeridian interactions. 

## Figures and Tables

**Table 1:** Mean current level change and normalizing ability after PLB irradiations.

	Before PLB (mean ± SD^#^)	After PLB (mean ± SD)	Statistic calculation *P* value by *t*-test
Mean current level of 24 meridian channels (candidates number = 76)	33.2 ± 15.9	29.6 ± 18.1	0.054 (>0.05)
Positive current value above mean current level (sampled meridian number = 69) for testing the normalizing ability of PLB irradiations on extremely high/moderately high current level (HCLMM)	22.8 ± 12.5	7.67 ± 13.8	<0.0001*
Negative current value below mean current level (sampled meridian number = 78) for testing the normalizing ability of PLB irradiations on extremely low/moderately low current level (LCLMM)	−25.8 ± 9.53	−7.30 ± 17.3	<0.0001*

^#^SD is standard deviation. *Mean significant difference.

**Table 2:** The overall percentage of one specific meridian channel indirectly affecting another meridian channel according to the change of current level over 50% or change of its current level category^#^.

Meridian channel Treated by PLB (candidates number)	Positive influence on “lung”	Positive influence on “pericardium”	Positive influence on “heart”	Positive influence on “spleen”	Positive influence on “liver”
“Gall bladder” (24)	71%	54%	50%	—	—
“Lung” (7)	—	—	—	71%	86%
“Triple energizer” (5)	—	—	—	60%	—

^#^Current level categories of extremely high current level, moderately high current level, normally high current level, extremely low current level, moderately low current level, and normally low current level.

**Table 3:** Meridian-related publications (2000–2013) that used evidence-based scientific methods.

Researchers	Method	Finding or hypothesis
Zhang et al. [30]	Single-power alternating current (SPAC) instrument	The subcutaneous impedance is lower at the low-impedance points as measured by the SPAC, two-electrode method.
Langevin and Yandow [31]	Ultrasound and postmortem tissue sections	The sites of acupuncture points are at locations of intermuscular or intramuscular connective tissue planes
Ahn et al. [32]	Ultrasound and electrical impedance instrument	The acupuncture point probably located on the skin overlying the fascial plane separating the muscles.
Brătilă and Moldovan [33]	Harmonic medicine: harmonic sounds stimulate the lung (LU; shoutaiyin feijing) and kidney (KI; zushaoyin shenjing) meridians	Resonance can be developed using music to stimulate acupuncture points. In this kind of acupunctural stimulation, a symphony may act and play a listening role.
Choi and Soh [34]	Optical fiber from a tungsten-halogen lamp	Light propagates more efficiently along the meridian than the reference path, demonstrating a 20% or greater difference among all tested participants.
Schlebusch et al. [35]	(1) Moxibustion (2) Similar light stimulation (3) Infrared detector within 3–5 *μ*m range	Light channels appear within the body, seemingly identical to meridians.
Schlünzen et al. [36]	Positron emission tomography (PET); Cerebral blood flow in healthy humans	The penetration of the skin by using needles affects the medial frontal gyrus, whereas acupuncture of the LI-4 influences the putamen.
de Souza et al. [37]	Bioavailability of radio-pharmaceutical sodium pertechnetate	Uptake of the radiopharmaceutical in organs.
Zhang et al. [38]	Highly sensitive CO_2_ instruments	The high correlation of transcutaneous CO_2_ emissions along the meridian may illustrate that the metabolism on the meridian has similar changes or relationships. A strong correlation of energy metabolism activity exists among the body surfaces along the meridian, and an even stronger correlation exists among the acupoints on the meridian.
Lee et al. [39]	Optimal stimulator frequency of 40 Hz through the pericardium meridian; Hydrodynamic analysis	The mean transfer speed in the meridian (4 m/s) was significantly lower than it was in the adjacent control region (8.5 m/s, *P* < 0.001). Significant differences existed between the meridian and control points in attenuation rate (*P* < 0.001) and peak amplitude (*P* < 0.001). This implies that the composition of the meridian differs from that of the adjacent control regions.
Zhang et al. [40]	(1) Hydromechanic model (a) Guyton's method (b) Single pressure transducer (c) Two pressure transducers provided more stable measurement (2) The transmission of interstitial fluid pressure wave (3) Presentation of the channel by isotopic migration	The findings support the hypothesis that the interstitial fluid channels form the physiological and morphological basis of the acupuncture meridians described in detail by the ancient Chinese more than 2000 years ago.
